# Secular Changes in the Age of Menarche of Rural and Urban Girls from an Industrial Region of Poland in Relation to Family Structure

**DOI:** 10.3390/ijerph19148692

**Published:** 2022-07-17

**Authors:** Jarosław Domaradzki, Teresa Sławińska, Małgorzata Kołodziej, Zofia Ignasiak

**Affiliations:** Department of Biostructure, Wroclaw University of Health and Sport Sciences, al. I. J. Paderewskiego 35, 51-612 Wrocław, Poland; jaroslaw.domaradzki@awf.wroc.pl (J.D.); teresa.slawinska-ochla@awf.wroc.pl (T.S.); zofia.ignasiak@awf.wroc.pl (Z.I.)

**Keywords:** rate of sexual maturation, age of menarche, secular trends, urbanization factor, family structure

## Abstract

Purpose: The consequence of better living conditions for every subsequent generation is the phenomenon of change in the rate of maturation and body dimensions. The aim of this study was to evaluate the intergenerational changes in the age of menarche of girls living in rural and urban communities in the industrial region of Poland using samples from two different centuries and to determine the relationship between family structure (two-parent and single-parent) and sexual maturation of the girls in both environments. Methods: The study included 3643 rural and urban girls aged 7–16 from southwestern Poland (Copper Basin). The research was cross-sectional in each environment around 2000 and 2010, and it was carried out twice. Two types of family structure were taken into account: two-parent and single-parent families. The median age of menarche and odds ratio (OR) of menarche depending on the place of residence and family structure were calculated. The status quo method was used when assessing the age of menarche. Results: In both groups, the age of menarche significantly decreased during the decade. The median age of menarche in the urban girls was lower compared with their rural peers, but a significant difference was found only during the second examination (a decade after the first examination). There were no significant changes in menarche during the decade regarding family structure (neither in girls from two-parent families nor those from single-parent families), except in rural girls from two-parent families. Comparing the median age of menarche of girls from two-parent families with girls from single-parent families (urban and rural) showed lower median values in the girls from two-parent families, but no differences were significant. Conclusions: The acceleration of the maturation rate over the last decade has been observed among both rural and urban girls. Environmental differences in maturation rates between rural and urban girls increased over the course of the decade, and the difference between the rural and urban girls’ age of menarche was statistically significant in the second examination (around 2010).The living conditions related to family structure did not significantly affect the timing of menarche.

## 1. Introduction

Auxological research, when periodically repeated, can help track secular trends. Such inquiries can include, among others, studies on the rising rates of precocious puberty, the gradual increase in mean body height and mass, or shifts in body composition in subsequent generations. The above trends are particularly evident in the populations of developing countries that have not yet reached full economic maturity, as their mechanisms are based on the influence of modern civilization and improvements in general living conditions. The lack of these trends is regarded as a measure of a country’s economic and social stability [1].

In fact, the phenomenon of favorable intergenerational change is now being explained by the ability of future generations to better fulfill their “biological potential” primarily as the result of better living conditions [1,2]. The fact that trends such as accelerated sexual maturation and increased stature are still ongoing in Poland and in ever-younger age groups is a testament to the still large developmental potential of Poland’s current and future generations [3,4]. For this reason, the age of menarche and basic somatic characteristics (body height and mass), including height mass indexes (e.g., BMI), are treated as biological markers, since they are regarded to be those traits most sensitive to changes in living conditions [5,6,7,8].

However, the relationships between environmental factors and the timing of puberty are not as clear. Accelerated sexual maturation may be caused by an improvement in living conditions, but it can also be the result of increased stress or exposure to toxins [9]. On the one hand, studies have documented social gradients in the onset of puberty, similar to what was observed in the case of somatic characteristics. Girls from families with a higher socioeconomic status (measured by factors such as parental education or economic standing in the area where they live) were found to mature faster [1,2,7]. On the other hand, a number of studies indicated that accelerated maturation may be caused by certain mental stressors, such as family conflict or an absent parent [10,11,12,13]. It was found that the stress connected with living in a nontraditional family structure can accelerate the onset of puberty while inhibiting physical development [14,15]. At the same time, some studies have also indicated that accelerated puberty may be caused by pollutants, either industrial or chemical, including exposure to artificial fertilizers, pesticides, cosmetics, or paints [9,16,17]. Other studies, however, suggest that elevated blood lead levels delay the rate of sexual maturation in children [18].

Current studies on the age of menarche of female residents in the Lower Silesia region in Poland more often concern rural samples [19]. However, there is a lack of studies comparing rural and urban girls from the industrial region and, specifically, the assessment of secular changes at the turn of the 20th and 21st centuries. The ambiguous nature of the presented results raises more questions than answers, pointing to the need for in-depth analyses of the impact of socioeconomic and ecological factors on the sexual maturation of girls.

The aim of this study was to assess the rate of sexual maturation in girls living in the rural and urban industrial areas in southwestern Poland. In particular, our goal was to (1) assess the menarche age of girls living in rural and urban areas and (2) determine the relationship between family structure (two-parent and single-parent) and the level of sexual maturation of girls from both backgrounds.

## 2. Materials and Methods

### 2.1. Characteristics of the Population Sample

The research was a cross-sectional study carried out in the industrial region of southwest Poland. Children from eight villages (Brzeg Głogowski, Kotla, Kromolin, Nielubia, Rosochata, Pielgrzymka, and Spalona) and the town of Polkowice were examined. The study of rural girls was carried out in 1998 and 2001 (cohort P1) and in 2007 and 2010 (cohort P2). The study of urban girls was conducted in 1999 and 2002 (cohort P1) and in 2008 and 2011 (cohort P2). There were different participants in all examinations. To obtain a larger number of participants, we combined the participants examined in 1998 and 2001, 1999 and 2002, 2007 and 2010, and 2008 and 2011. Thus, initially, there were four study cohorts. Data were collected from students present at school on the day of the exam and who had their parents’ consent to participate in the study (84–87.5% of school children). In total, 1615 girls living in the countryside and 2028 girls living in an urban environment were examined. The age of the participants ranged from 7 to 16 years. The numbers of the rural and urban girls from both study periods for the earlier (P1) and later (P2) cohorts are presented in Table 1. Next, the participants were divided into subgroups based on family type (one-parent and two-parent).The numbers of the rural and urban girls from both study periods and the two types of families are presented in Table 2.

Demographic data not presented in the main text (published elsewhere in Slawinska et al., 2012 [19]) showed that the majority of the rural girls (examined in both periods) came from blue collar (mining or industrial workers) or farming families with primary or vocational education. Only a few of the rural girls’ parents completed secondary or higher education. The families of the city girls (examined in both periods) were comparable in terms of education (differences in proportions were not statistically significant) [20].

The research was funded under grants from the Ministry of Science: P0D07508, P0D01226, and P05D00226. The study protocol was approved by the Senate Research Ethics Committee of Wroclaw University of Health and Sport Sciences and was consistent with the ethical requirements for human experimentation under the Declaration of Helsinki. The children and their guardians were informed about the purpose and methods of the research, all the procedures, and the experimental risks. The legal guardians gave written informed consent for each child to participate in the study. All children voluntarily participated in the tests.

### 2.2. Study Methods

In this study, the chronological age and the age of menarche were taken into account. Information on the time of menarche was collected using the status quo method, where each girl was asked directly if she had had her first menses. On this basis, the fraction of girls who had their first menses (or not) was determined for each age group. A questionnaire was also administered to determine the number of girls living in a two-parent or single-parent family (Table 2).

### 2.3. Statistical Analyses

The menarcheal age of the girls was analyzed in two research periods—earlier (cohort P1) and later (cohort P2)—and in two environments: rural and urban. Two types of families, two-parent and single-parent, were identified.

Probit analysis was used for each cohort (the girls from the earlier and later study periods) to estimate the median age of menarche and its variability using a 95% confidence interval. Two-way and three-way ANOVA with post hoc Tukey’s tests were used to assess the significance of the differences in chronological and menarcheal age between the groups.

Logistic regression was performed using the maximum likelihood estimation to estimate the odds ratio (OR) of menarche depending on age, place of residence, and family structure. The menarche status (0 = no, 1 = yes) was treated as the dependent variable. An odds ratio (OR), derived from logistic regression, with a value less than 1.0 indicated that the independent variables lessened the odds of the event occurring (in this case, the onset of menarche). When the OR exceeded 1.0, this indicated that the independent variables increased the likelihood of menarche occurring.

Statistical significance was set at *p* < 0.05. TIBCO Statistica 13.3.0 (StatSoft Poland, Kraków, Poland) was used for all calculations.

## 3. Results

The mean calendar age for the rural girls from P1 was 10.86 years (95%Cl: 10.72–11.00), and for the rural girls from P2, it was 11.73 years (95%Cl: 11.54–11.93). The mean calendar age for the urban girls from P1 was 10.95 years (95%Cl: 10.82–11.08), and for the girls from P2, it was 10.19 years (95%Cl: 10.02–10.35). The number of girls in the cohorts was not equal in size, and this fact might have affected the differences in the means for the calendar age for the girls in each cohort. Detailed comparisons of Tukey’s post hoc tests (derived from ANOVA tests) showed no significant difference between the ages of the rural and urban girls studied in P1 (*p* = 0.805), while the difference was significant in P2 (*p* < 0.01).

The median of menarche for the rural girls in P1 was 13.36 years (95%Cl: 13.00–13.72), and for the rural girls in P2, it was 12.92 years (95%Cl: 12.55–13.29). Acceleration of the onset of menstruation in rural girls was statistically significant (*p* < 0.001) (Table 3). The median of menarche for the urban girls in P1 was 13.12 years (95%Cl: 12.81–13.44), and for the urban girls in P2, it was 12.46 years (95%Cl: 12.27–12.65). The shift in timing of the first menstruation in the urban girls examined in P2 compared with the girls examined in P1 was statistically significant (*p* < 0.001).

The difference in the median of menarche between the rural and urban girls examined in P1 was not statistically significant (*p* = 0.390), while the menarche of the urban girls examined in P2 was significantly earlier compared with that of the rural girls examined in P2 (*p* < 0.001) (Table 3).

Analysis performed on the family structure was used to evaluate the influence of intra-family factors on the age of sexual maturation. Despite both urban and rural girls living in two-parent families being found to mature at an earlier age compared with girls living in single-parent families (both at the beginning and the end of the analyzed time span), the only significant difference was observed with the rural girls studied in P1 (Table 4).

The shift in timing during a decade (by subtracting the median age at which the girls were examined in P2 from P1)across the place of residence and family structure was larger for urban girls compared with the rural girls (in both single-parent and two-parent families). However, the differences were not statistically significant.

To assess the effect the place of residence and family structure had on the age of menarche and the change in both effects over a decade, logistic regression was performed separately for the cohorts from the first and second study periods (P1 and P2).

The strongest and statistically significant determinant of experiencing menarche, as was expected, was age (Table 5). Among both factors analyzed in this work, only the place of living factor was significant (P1: OR = 1.47, 95% CI: 1.09–1.98; P2: OR = 2.19, 95% CI: 1.37–3.50). Performing this analysis separately for girls surveyed at the beginning and end of the analyzed timeframes showed that the relative influence of the place of residence on the likelihood of menarche had increased over time. The girls living in urban areas examined in P2 had over twofold higher odds of having attained menarche than their rural peers (Table 5).

## 4. Discussion

The aim of our work was to study the secular changes in the age of menarche regarding the place of residence and type of family. The results confirmed a significant acceleration over a decade in girls from both agglomerations. Comparing urban girls with rural girls showed a significant difference only between the girls examined in P2 (urban girls matured faster than rural girls), whereas there were no significant changes in menarche over a decade regarding family structure (neither in girls from two-parent families nor those from single-parent families), except in rural girls from two-parent families. Comparing the median age of menarche of girls from two-parent families with girls from single-parent families (urban and rural) showed lower median values in the girls from two-parent families, but any differences were not significant. The girls in the present study examined in P1 matured later compared with the national average in 1999 (median age of menarche: 12.8 years) [21], while the girls examined in P2 matured earlier when compared with a nationwide sample examined in 2012 (average median: 13.0 years; median for urban girls: 13.2 years, median for rural girls: 13.3 years) [1,21].

Given the strong relationships between socioeconomic and ecological factors with the rate of maturation and age of menarche, the environmental changes that took place over the 1990s and 2000s in Poland were found to not negatively affect the rate of maturation. In this respect, the transformations that took place in the environments of the surveyed girls in the decade from 2000 to 2010 should be viewed positively. The results obtained from our study are generally in accordance with the newest results in Poland observed by Gomuła and Kozieł [1], Bartkowiak et al. [3], and international studies [2,7]. On the contrary, some studies documented slower growth and acceleration in environments impacted by unfavorable factors [11,13]. Danker-Hopfe and Hulanicka [9] observed an acceleration of maturation related to harmful chemical substances (pollutants, pesticides, etc.) present in polluted areas. Exposure to toxic substances can accelerate puberty as a biological phenomenon related to the need to extend the duration of a species in the event of environmental threats.

Based on the present results, menarche was not closely related to the socioeconomic conditions of the girls’ families. On the contrary, several authors suggested the effects of emotional stimuli on the disruption in the concordant development of children and adolescents [10,22,23]. Our observations did not show earlier maturation in girls from two-parent families compared with one-parent families, except in rural girls examined at the beginning of the 2000s. Further studies on the effects of the complex interactions between some intra-family factors that shape a child’s emotional state and those which are tied to the socioeconomic status of single-parent families on the age of menarche are needed, where the influence of the social and family environmental factors may change more over time.

Lifestyle changes, especially nutrition, socioeconomic factors, local environmental variables, and endogenous elements, are those that modify sexual maturation [1,2,24]. The observed secular trends of menarcheal age (fastening maturation), both in the urban and rural cohorts, increase the risk of many health problems in women such as obesity, diabetes, cardiovascular disease, and breast cancer [25], so it is important to look into the factors that may influence the age of menarche. The urgency of researching this subject matter has been highlighted by the increasingly more influential impact of environmental factors, particularly the place of residence.

The strengths of this study were (1) a large group of participants in both the rural and urban environments, (2) homogeneous hazards from the industrial environment during the studied decade, (3) favorable economic situations of the surveyed families (employment in the mining industry of parents living in the city and countryside), (4)repeated studies, and (5) a permanent and experienced research team in both examinations. One of the limitations is the status quo method used to estimate the age of menarche. Another limitation is the fact that only two factors that affect menarche were studied. Due to the possible effects of interactions between many factors, future research should include a multi-dimensional approach while taking into account additional environmental and endogenous variables and in-depth analyses. Another limitation was very unequal proportions between the number of girls from two-parent families and single-parent families. The bias might have resulted in a lack of significance in the comparisons.

## 5. Conclusions

The acceleration of the maturation rate over the last decade has been observed among both rural and urban girls. Environmental differences in maturation rates between rural and urban girls increased over the decade. The examined girls from urban places of residence attained menarche significantly earlier compared with the girls residing in rural communities examined in P2. It can be suggested that different factors linked to larger agglomerations can accelerate maturity. The importance of these factors has grown over time (in contrast to the lack of significant differences between the urban and rural girls surveyed in P1). Therefore, the role the place of residence variable plays in stimulating the time of sexual maturation increased in the analyzed decade, whereas the living conditions related to family structure did not significantly affect the timing of menarche. However, continuing research on the influence of both analyzed factors on the rate of maturation is suggested. Despite the lack of significant differences, some regularities in the rate of maturation were observed: the age of menarche decreased in rural girls from single-parent families, rural girls from two-parent families, and urban girls from single-parent families, with the fastest maturation being for urban girls from single-parent families. It is worth assessing whether both factors affect the timing of menstruation additively or not.

## Figures and Tables

**Table 1:** The number of rural and urban girls grouped by age for each study period.

	Age (Years)										Number of Girls (*n*)
Place of Residence—Study Period	7	8	9	10	11	12	13	14	15	16	
rural—P1 cohort (1998 and 2001)	109	111	129	143	139	130	129	105	55	15	1065
rural—P2 cohort (2007 and 2010)	27	52	53	62	48	64	76	81	77	10	550
urban—P1 cohort (1999 and 2002)	61	123	212	211	142	186	145	142	57	12	1291
urban—P2 cohort (2008 and 2011)	88	116	126	107	103	76	39	40	29	13	737
Total	3643										

**Table 2:** The number of rural and urban girls from two-parent or single-parent families for each study period.

Place of Residence—Study Period	Family Structure (*n*)			
	Two-Parent	Single-Parent	Total	Percent of Girls from Single-Parent Families (%)
rural—P1 cohort (1998 and 2001)	974	91	1065	8.55
rural—P2 cohort (2007 and 2010)	467	83	550	15.10
urban—P1 cohort (1999 and 2002)	1132	159	1291	12.32
urban—P2 cohort (2008 and 2011)	638	99	737	13.43
Total	3211	432	3643	11.86

**Table 3:** Medians with 95% confidence intervals, showing χ^2^ of the fitted probit models for estimated ages of menarche and *p*-values between groups(rural and urban, P1 and P2 cohorts).

Place of Residence—Study Period	Age of Menarche (Years)			Statistical Analysis	Between Periods	Between Rural and Urban Girls in Periods
	Median	95% CI		χ^2^ of the Model (*p*)	*p*	*p*
rural—P1 cohort (1998 and 2001)	13.36	13.00	13.72	31.37 (<0.001)	<0.001	P1 rural and urban 0.390
rural—P2 cohort (2007 and 2010)	12.92	12.55	13.29	21.04 (0.013)		
urban—P1 cohort (1999 and 2002)	13.12	12.81	13.44	29.80 (0.001)	<0.001	P2 rural and urban <0.001
urban—P2 cohort (2008 and 2011)	12.46	12.27	12.65	23.93 (0.011)		

**Table 4:** Medians with 95% confidence intervals and χ^2^ of the fitted probit models for estimated ages of menarche for rural and urban girls according to study period(P1 and P2 cohorts) from two-parent and single-parent families.

Place of Residence—Study Period	Family Structure	Age of Menarche (Years)			Statistical Analysis	Between Periods	Between Two-Parent and Single-Parent (Rural and Urban, P1 and P2)
		Median	95% CI		χ^2^ of the Model *(p)*	*p*	*p*
rural—P1	Two-parent	13.30	12.89	13.71	38.60 (<0.001)	0.010	ns
	Single-parent	13.86	13.27	14.45	1.75 (0.995)		
urban—P1	Two-parent	13.09	12.94	13.24	16.05 (0.066)	ns	
	Single-parent	13.37	12.84	13.90	11.67 (0.232)		
rural—P2	Two-parent	12.87	12.41	13.33	26.75 (0.002)	ns	
	Single-parent	13.23	12.68	13.78	1.95 (0.992)		
urban—P2	Two-parent	12.40	12.21	12.59	2.29 (0.986)	ns	
	Single-parent	12.68	12.07	13.29	4.59 (0.868)		

ns—not statistically significant at *p* < 0.05.

**Table 5:** Results of the logistic regression on the odds ratio of menarche occurring.

	Predictor	Coefficient Estimate	SE	OR (95% CI)	Wald Statistic	*p*-Value
P1 period (1998–2002)	Intercept	−19.19	0.93		421.47	<0.001
	Age	1.41	0.07	4.10 (3.59–4.68)	435.89	<0.001
	Urban residence	0.38	0.15	1.47 (1.09–1.98)	6.45	0.011
	Two parents	0.38	0.24	1.46 (0.90–2.34)	2.38	0.123
P2 period (2007–2011)	Intercept	−20.96	1.41		221.77	<0.001
	Age	1.59	0.10	4.92 (4.02–6.03)	238.95	<0.001
	Urban residence	0.78	0.24	2.19 (1.37–3.50)	10.72	0.001
	Two parents	0.33	0.30	1.39 (0.77–2.52)	1.21	0.271

SE = standard error; OR = odds ratio; 95% CI = confidence interval.

## Data Availability

The data presented in this study are available on request from the corresponding author. The data are not publicly available due to ethical restrictions.
